# The Dual Functions of Andrographolide in the Epstein–Barr Virus-Positive Head-and-Neck Cancer Cells: The Inhibition of Lytic Reactivation of the Epstein–Barr Virus and the Induction of Cell Death

**DOI:** 10.3390/ijms242115867

**Published:** 2023-11-01

**Authors:** Chukkris Heawchaiyaphum, Praphatson Malat, Chamsai Pientong, Sittiruk Roytrakul, Yodying Yingchutrakul, Sirinart Aromseree, Supawadee Suebsasana, Panupong Mahalapbutr, Tipaya Ekalaksananan

**Affiliations:** 1 Department of Microbiology, Faculty of Medicine, Khon Kaen University, Khon Kaen 40002, Thailand; chukheaw@tu.ac.th (C.H.); phatson88@gmail.com (P.M.); 2 Department of Biotechnology, Faculty of Science and Technology, Rangsit Center, Thammasart University, Pathum Thani 12120, Thailand; 3 Faculty of Agriculture and Technology, Nakhon Phanom University, Nakhon Phanom 48000, Thailand; sirinar@kku.ac.th; 4 HPV & EBV and Carcinogenesis Research Group, Khon Kaen University, Khon Kaen 40002, Thailand; 5 Functional Ingredients and Food Innovation Research Group, National Center for Genetic Engineering and Biotechnology, National Science and Technology Development Agency, Pathum Thani 12120, Thailand; sittiruk@biotec.or.th (S.R.); yodying.yin@nstda.or.th (Y.Y.); 6 Department of Pharmaceutical Sciences, Faculty of Pharmacy, Rangsit Center, Thammasat University, Pathum Thani 12120, Thailand; hnungnet@yahoo.com; 7 Department of Biochemistry, Faculty of Medicine, Khon Kaen University, Khon Kaen 40002, Thailand; panupma@kku.ac.th

**Keywords:** andrographolide, EBV, EBV lytic reactivation, HDAC5, MEF2D, SP1, SP3, cell death, necroptosis

## Abstract

Andrographolide, a medicinal compound, exhibits several pharmacological activities, including antiviral and anticancer properties. Previously, we reported that andrographolide inhibits Epstein–Barr virus (EBV) lytic reactivation, which is associated with viral transmission and oncogenesis in epithelial cancers, including head-and-neck cancer (HNC) cells. However, the underlying mechanism through which andrographolide inhibits EBV lytic reactivation and affects HNC cells is poorly understood. Therefore, we investigated these mechanisms using EBV-positive HNC cells and the molecular modeling and docking simulation of protein. Based on the results, the expression of EBV lytic genes and viral production were significantly inhibited in andrographolide-treated EBV-positive HNC cells. Concurrently, there was a reduction in transcription factors (TFs), myocyte enhancer factor-2D (MEF2D), specificity protein (SP) 1, and SP3, which was significantly associated with a combination of andrographolide and sodium butyrate (NaB) treatment. Surprisingly, andrographolide treatment also significantly induced the expression of DNA Methyltransferase (DNMT) 1, DNMT3B, and histone deacetylase (HDAC) 5 in EBV-positive cells. Molecular modeling and docking simulation suggested that HDAC5 could directly interact with MEF2D, SP1, and SP3. In our in vitro study, andrographolide exhibited a stronger cytotoxic effect on EBV-positive cells than EBV-negative cells by inducing cell death. Interestingly, the proteome analysis revealed that the expression of RIPK1, RIPK3, and MLKL, the key molecules for necroptosis, was significantly greater in andrographolide-treated cells. Taken together, it seems that andrographolide exhibits concurrent activities in HNC cells; it inhibits EBV lytic reactivation by interrupting the expression of TFs and induces cell death, probably via necroptosis.

## 1. Introduction

The Epstein–Barr virus (EBV), a member of the gamma-herpesvirus family, is an enveloped virus containing a linear, double-stranded DNA genome. EBV is ubiquitous, infecting more than 90% of the world’s adult population, with transmission mostly mediated via the oral route. The primary infection by EBV occurs in the oral cavity. EBV infection is closely associated with various types of human malignancies, including Burkitt’s lymphoma, Hodgkin’s lymphoma, NK/T-cell lymphoma, nasopharyngeal carcinoma (NPC), EBV-associated gastric carcinoma (EBVaGC), breast carcinoma, and oral squamous cell carcinoma (OSCC) [1,2,3].

EBV exhibits two alternative phases in the course of infection: latent and lytic. During the lytic phase, new virions are produced and released from the host cell to infect new target cells. EBV lytic reactivation is mediated by both host and virus factors. The molecular mechanism underlying the regulation of EBV lytic reactivation has yet to be fully elucidated. Accumulating evidence has demonstrated that the host master transcriptional regulators of cell differentiation, such as BLIMP1 and KLF4, and transcription factors (TFs), such as ATF, Sp1/3, MEF2D, XBPs, CREB family members, C/EBP family members, AP1, and HIF1-α, can activate the Zta promoter (Zp) and Rta promoter (Rp) of EBV by binding to the responsive elements located on these promoters. On the other hand, host TFs, such as YY1, ZEB1, and ZEB2, can suppress the activation of Zp and Rp [4,5,6,7,8,9,10]. 

Recent evidence suggests that both latent EBV infection and lytic EBV infection contribute to the genesis of EBV-associated malignancies. BZLF1 is a lytic gene of EBV that plays a critical role in transcriptional transactivation to regulate the switching of EBV from latency to lytic replication [7]. The Zta, a product of the BZLF1 gene, contributes to the oncogenesis of EBV-associated malignancies by inducing genome instability, inflammation, cell proliferation, and inhibiting cell death through the activation of several signaling pathways [11,12,13]. In addition, Rta, a product of the BRLF1 gene, can also promote the oncogenesis of EBV-associated malignancies by inducing cell migration via the activation of the IL6/JAK/STAT3 signaling pathway [14]. Moreover, BRLF1 can also activate the expression of LMP1 during EBV lytic replication in epithelial cells. The expression of LMP1 enhances the promotor activity of Zp and Rp [15,16,17,18,19,20]. It is well documented that LMP1 plays critical roles in EBV-mediated malignant transformation, including resistance to apoptosis and increased angiogenesis, invasion, and metastasis in EBV-harboring cancer cells through the activation of oncogenic signaling pathways, such as the NF-κB, PI3-K/Akt and JAK/STAT pathways [1,21,22,23,24].

As mentioned previously, the lytic infection of EBV is not only associated with the transmission of virus, but it also plays a role in the development of various cancers. Therefore, the inhibition of lytic reactivation by using natural compounds is valuable for the prevention and treatment of EBV-associated diseases. Andrographolide is a medicinal compound that is extracted from the herb *Andrographis paniculata* (Burm. f.) Nees. This compound exhibits antiviral activity, including severe acute respiratory syndrome coronavirus 2 (SARS-CoV-2) and EBV [25,26]. Andrographolide inhibits EBV replication by inhibiting the expression of EBV lytic proteins, including Rta, Zta, and EA-D, during lytic activation in P3HR1 cells and thus inhibits the promoter activities of Zta and Rta [27]. Similarly, our previous study also demonstrated that andrographolide inhibits lytic gene expression and hinders the production of viral particles [28,29]. However, the underlying molecular mechanism through which andrographolide performs these functions in each type of epithelial malignancy is still unknown. Therefore, we aimed to fill this knowledge gap using EBV-positive head-and-neck cancer (HNC) cell lines. Here, we demonstrate for the first time that andrographolide exhibits two concurrent activities in such cells. Firstly, it limits EBV lytic reactivation through the inhibition of the expression of relevant EBV genes and EBV virion production via the inhibition of TFs, MEF2D, SP1, and SP3, possibly through epigenetic mechanisms. Secondly, andrographolide also induces cell death, probably via necroptosis in HNC cells.

## 2. Results

### 2.1. Andrographolide Modulates the Pattern of Protein Expression in the EBV-Infected NPC Cell Line

**Figure 1:**
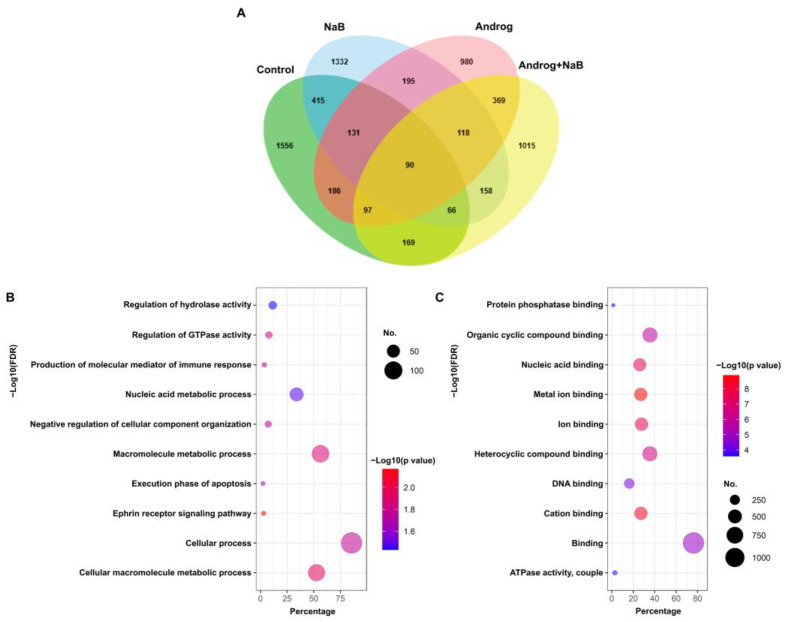
Andrographolide alters the proteomic profile of an EBV-infected NPC cell line (HONE1-EBV): (**A**) Venn diagram for proteins expressed in HNC cells treated with DMSO (control), NaB alone, andrographolide alone, or a combination of andrographolide and NaB. GO enrichment analysis of differentially expressed proteins was carried out using DAVID. The ten most significantly (*p* < 0.05) enriched GO terms in biological process (**B**) and molecular function (**C**) are presented as bubble diagrams. The Y-axis reveals the top ten functional enrichment results. The X-axis indicates the percentage of genes involved in each biological process or molecular function. The color represents the *p* value: a range from blue to red indicates a low to high *p* value, respectively. The size of each bubble indicates the gene numbers involved in each biological process or molecular function. NaB: sodium butyrate, Androg: andrographolide.

To identify the proteins that were markedly differentially expressed in the EBV-infected NPC cell line, HONE1-EBV, we analyzed the proteomic profiles of the treated cells in the four different treatments. A Venn diagram was constructed, which is shown in Figure 1A. In total, 1015 proteins were expressed only in the cells treated with andrographolide in combination with NaB (Figure 1A). In addition, gene ontology (GO) enrichment analysis was conducted on these 1015 proteins using the Database for Annotation, Visualization, and Integrated Discovery (DAVID) as a tool for annotating gene functions. The 1015 proteins analyzed in this study were significantly enriched in biological processes, such as cellular processes (85.5%) and macromolecule metabolic processes (56.4%), among others (Figure 1B). In addition, proteins for binding (76.2%), organic cyclic-compound binding (35.7%), etc., were significantly enriched in the molecular function category (Figure 1C). 

### 2.2. Andrographolide Inhibits EBV Lytic Reactivation in HNC Cell Lines

**Figure 2:**
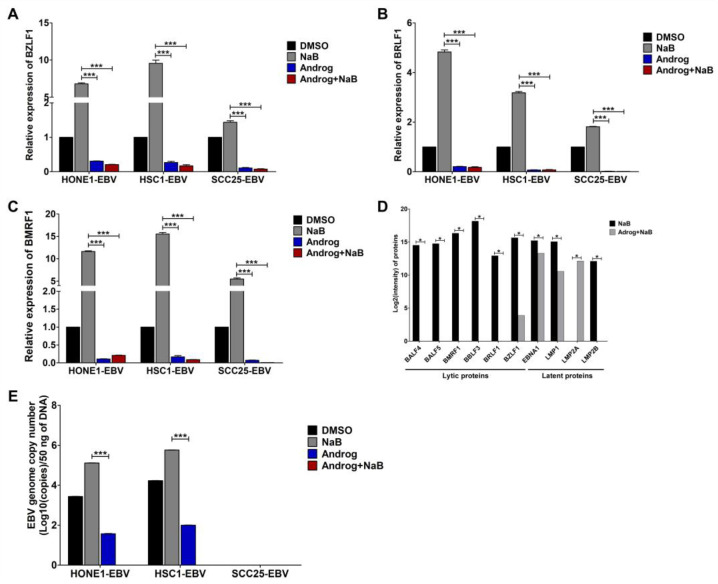
Andrographolide inhibits EBV lytic reactivation in HNC cell lines. Cells were treated with DMSO (control), NaB, andrographolide, or a combination of andrographolide and NaB for 48 h, and the expression of EBV lytic genes, including BZLF1 (**A**), BRLF1 (**B**), and BMRF1 (**C**), was examined using qRT-PCR. The expression of EBV lytic and latent proteins was examined via LC-MS/MS (**D**). The EBV genome’s copy number was quantified using qPCR (**E**). NaB: sodium butyrate, Androg: andrographolide. * *p* < 0.05, *** *p* < 0.001.

Our previous study demonstrated that andrographolide inhibits EBV lytic reactivation through the suppression of lytic protein expression and the inhibition of EBV virion production [28,29]. In this study, we further examined the inhibitory effect of andrographolide on the inhibition of EBV lytic reactivation in HNC cell lines via the treatment of EBV-positive HNC cell lines (HONE1-EBV, SCC25-EBV, and HSC1-EBV) with NaB alone, andrographolide alone, or andrographolide combined with NaB. We observed a significant decrease in the expression of EBV lytic genes in HNC cell lines treated with andrographolide and the combination of andrographolide and NaB when compared with NaB treatment (Figure 2A–D). 

Additionally, the copy number of the EBV genome was estimated via qPCR. Consistently, the EBV genome’s copy number was significantly lower in EBV-positive HNC cell lines treated with andrographolide alone and the combination of andrographolide and NaB than in those treated with NaB only (Figure 2E). Therefore, these results indicate that andrographolide inhibits EBV lytic reactivation in HNC cells through the suppression of EBV lytic protein and virion production.

### 2.3. Andrographolide Inhibits EBV Lytic Reactivation via the Dysregulation of TFs 

**Figure 3:**
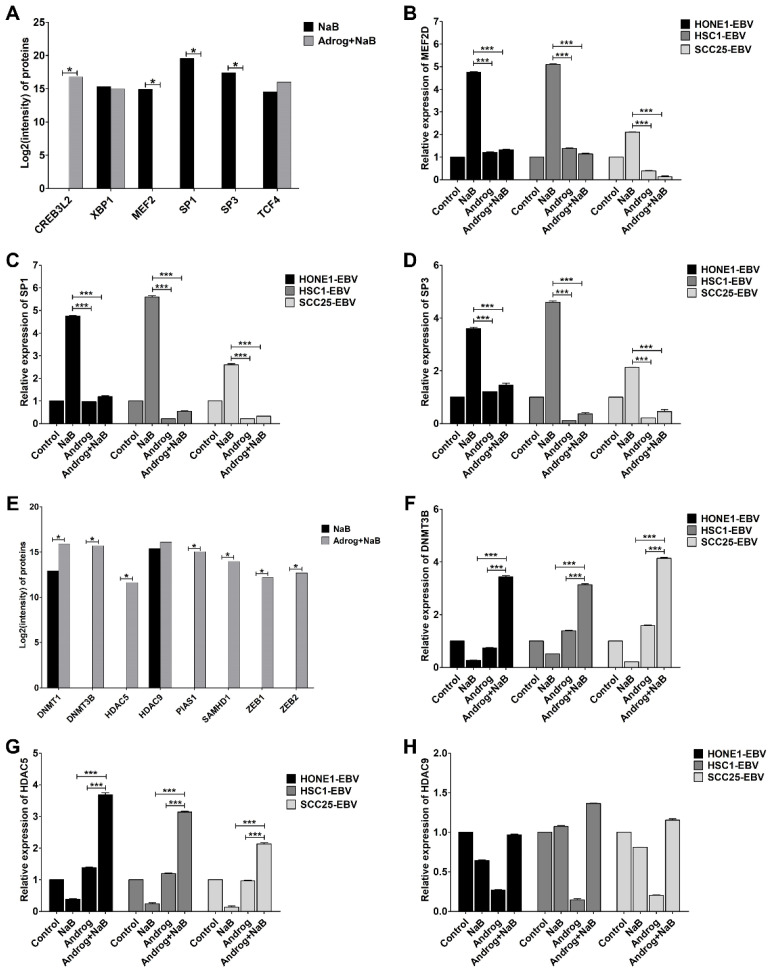
Andrographolide inhibits the expression of TFs related to EBV lytic reactivation. Cells were treated for 48 h with NaB, andrographolide, or a combination of andrographolide and NaB. The expression of TFs was examined via LC-MS/MS (**A**). The expression of MEF2D (**B**), SP1 (**C**), and SP3 (**D**) was determined using qRT-PCR. The expression of repressors was examined via LC-MS/MS (**E**), and the expression of DNMT3B (**F**), HDAC5 (**G**), and HDAC9 (**H**) was examined using qRT-PCR. NaB: sodium butyrate, Androg: andrographolide. * *p* < 0.05, *** *p* < 0.001.

Accumulating evidence has demonstrated that the activation of EBV lytic replication can be initiated or inhibited by TFs, both transcriptional transactivators and transcriptional repressors. Hence, we hypothesized that andrographolide modulates the expression of these TFs to regulate EBV lytic reactivation in HNC cells. To test our hypothesis, liquid chromatography with tandem mass spectrometry (LC-MS/MS) was used to examine the expression levels of TFs. Interestingly, MEF2D, SP1, and SP3 proteins were significantly decreased in EBV-positive HNC cells treated with the combination of andrographolide and NaB when compared with NaB treatment alone (Figure 3A). Consistent with this finding, the expression of MEF2D, SP1, and SP3 genes was significantly decreased in EBV-positive HNC cell lines treated with the combination of andrographolide and NaB when compared with NaB treatment alone (Figure 3B–D). In addition, we also examined the expression of transcriptional repressors via LC-MS/MS. As shown in Figure 3E, the expression of transcriptional repressors ZEB1, ZEB2, PIAS1, and SAMHD1 was significantly increased in EBV-positive HNC cell lines treated with the combination of andrographolide and NaB when compared with NaB treatment alone. Surprisingly, in cells subjected to the combination treatment, the expression of key molecules involved in epigenetic mechanisms, including DNA methyltransferase (DNMT) 1, DNMT3B, histone deacetylase (HDAC) 5, and HDAC9, was significantly greater (Figure 3E–H). Therefore, these results highlight the mechanism used by andrographolide to inhibit the lytic reactivation of EBV by modulating the expression of TFs and epigenetic changes.

### 2.4. Prediction of the Interaction of Histone Deacetylases (HDACs and TFs) Using Molecular Modeling and Docking 

**Figure 4:**
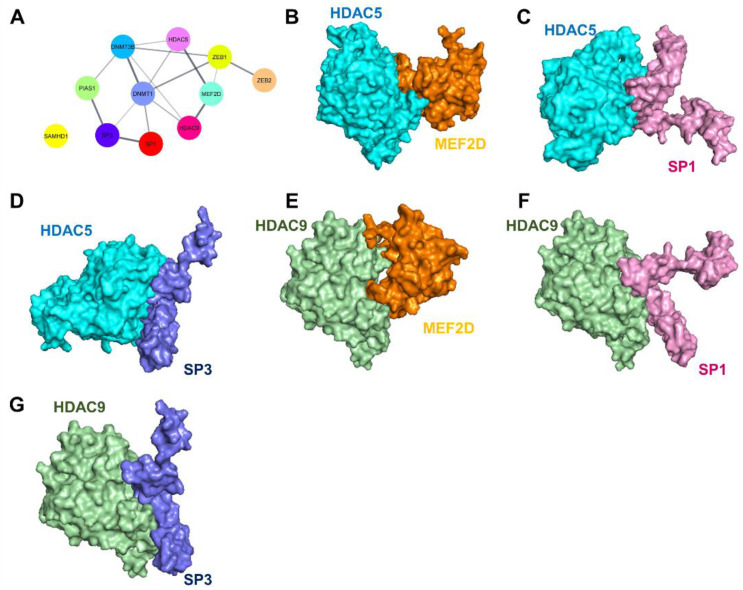
HDAC5 and HDAC9 can directly interact with MEF2D, SP1, and SP3. The PPI was constructed using the STRING database and Cytoscape software version 3.9.1 (**A**). The interactions of HDAC5/MEF2D (**B**), HDAC5/SP1 (**C**), HDAC5/SP3 (**D**), HDAC9/MEF2D (**E**), HDAC9/SP1 (**F**), and HDAC9/SP3 (**G**) were predicted using ClusPro and visualized with PyMOL and Discovery Studio 2021 software version 21.1.

It is well documented that HDACs regulate gene expression via deacetylation and that some HDACs can directly interact with target proteins [30,31]. Therefore, we further examined whether HDAC5 and HDAC9 can directly interact with MEF2D, SP1, and SP3 by using the PSOPIA web tool. The results showed that, based on protein sequences, both HDAC5 and HDAC9 could directly interact with MEF2D, SP1, and SP3, as evidenced by the Averaged One-Dependence Estimator (AODE) scores (Table 1). Consistent with this result, using the Search Tool for the Retrieval of Interacting Genes (STRING) database, we predicted that proteins related to the regulation of EBV lytic replication are able to interact with each other, except for SAMHD1 (Figure 4A). 

To confirm whether HDACs can directly bind with TFs, molecular modeling and docking simulation were performed. As expected, HDACs could bind to TFs via several electrostatic interactions and hydrogen bonds (Figure 4B–G and Tables S1 and S2). The coefficient weights of the lowest energy of the interactions of HDAC5/MEF2D, HDAC5/SP1, HDAC5/SP3, HDAC9/MEF2D, HDAC9/SP1, and HDAC9/SP3 were −765.9, −1155.5, −940.7, −815.3, −1019.8, and −1065.3, respectively. These results suggest that HDAC5 and HDAC9 can directly interact with MEF2D, SP1, and SP3, and thus they can inhibit their transactivation activity. 

### 2.5. Andrographolide Induces Cytotoxicity and Cell Death of HNC Cells

**Figure 5:**
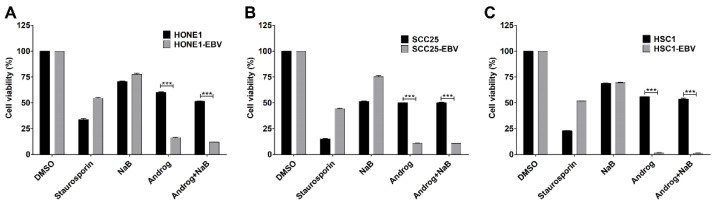
Andrographolide exhibits a strong cytotoxic effect on EBV-positive HNC cell lines. Cells were treated with DMSO (1% *v*/*v*), NaB (3 mM), andrographolide (HONE1 and HONE1-EBV: 36.3 μM; SCC25 and SCC25-EBV: 35.0 μM; and HSC1 and HSC1-EBV: 35.0 μM), or the combination of andrographolide and NaB for 48 h, and cytotoxicity in HONE1 (**A**), SCC25 (**B**), and HSC1 (**C**) was examined using CCK-8. Staurosporine (100 nM) was used as a positive control for cytotoxicity. *** *p* < 0.001.

To determine whether andrographolide is cytotoxic for HNC cells and induces cell death, the Cell Counting Kit-8 (CCK-8) was used to determine cytotoxicity in both EBV-positive and EBV-negative HNC cell lines. As shown in Figure 5, andrographolide treatment significantly reduced the cell viability of EBV-positive cell lines but not EBV-negative cell lines, when compared with the NaB treatment. This result suggests that andrographolide is cytotoxic for HNC cells and induces cell death, particularly in EBV-positive HNC cells.

**Figure 6:**
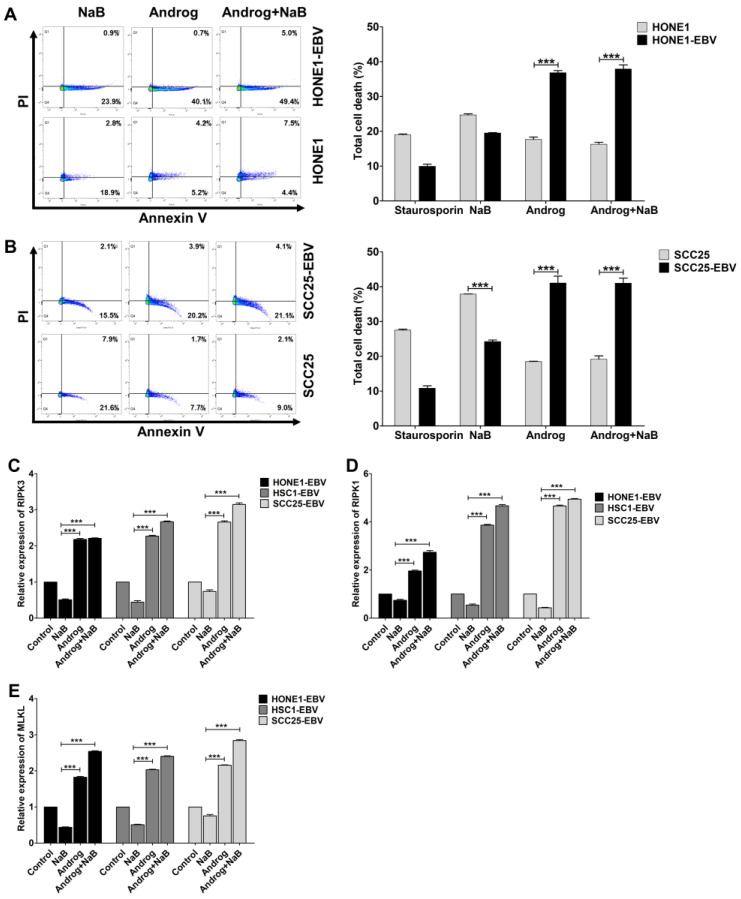
Andrographolide induces cell death via the necroptosis pathway. The effect of andrographolide on the induction of cell death in NPC (**A**) and OSCC (**B**) cells was assessed via flow cytometry. The expression levels of key mediators of necroptosis, namely RIPK1 (**C**), RIPK3 (**D**), and MLKL (**E**), were examined using qRT-PCR. Staurosporine was used as a positive control for cell apoptosis. *** *p* < 0.001.

To gain more insight into the functional pathway that was influenced by andrographolide treatment, we used the Kyoto Encyclopedia of Genes and Genomes (KEGG) analysis to investigate differentially expressed proteins. The result revealed that, in addition to apoptosis, the necroptosis pathway was enriched in andrographolide-treated cells when compared with NaB-treated cells. Furthermore, we confirmed the proteomic results via the determination of cell death using flow cytometry. As expected, andrographolide dramatically induced cell death in HNC cell lines, especially EBV-positive cell lines, via both necrosis and apoptosis when compared with NaB treatment (Figure 6A,B). 

Furthermore, we confirmed the expression of key molecules in the necroptosis pathway, namely RIPK1, RIPK3, and MLKL, via qRT-PCR. As expected, the expression of these genes was significantly greater in andrographolide-treated cells (Figure 6C–E). These results suggest that andrographolide induces cell death in HNC cells, probably via the necroptosis pathway.

**Figure 7:**
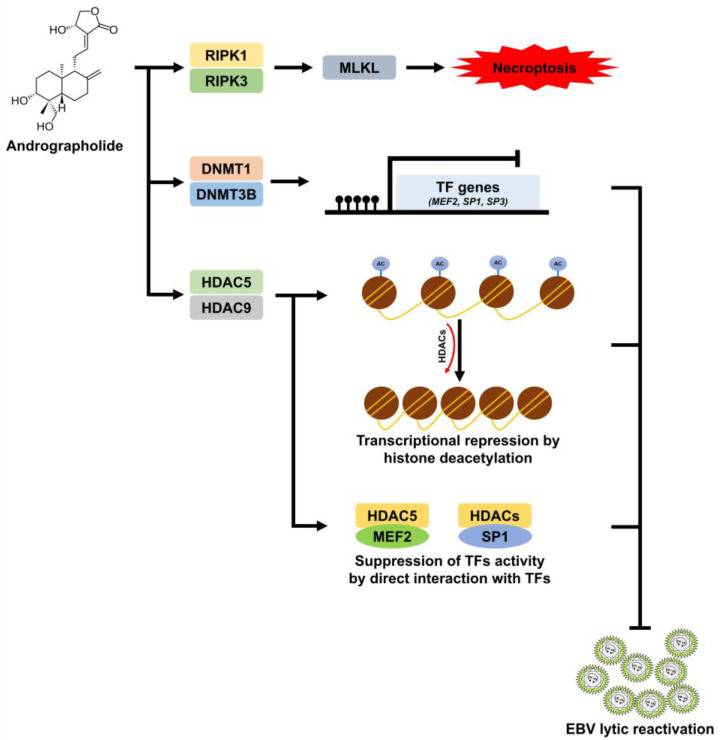
Possible mechanisms through which andrographolide inhibits EBV lytic reactivation and induces cell death in HNC cells. Andrographolide treatment induces the expression of the key mediators of necroptosis, namely RIPK1, RIPK3, and MLKL, resulting in cell death. In addition, andrographolide also induces the silencing of target genes, in particular TF genes (MEF2, SP1, and SP3), via the increased expression of DNMT1, DNMT3B, HDAC5, and HDAC9. HDAC5 and HDAC9 may directly interact with MEF2, SP1, and SP3, resulting in the inhibition of the transcriptional activity of these TFs.

Overall, we demonstrated, for the first time, that the mechanism through which andrographolide inhibits EBV lytic reactivation in HNC cells is the dysregulation of TFs through epigenetic mechanisms, namely DNA methylation and histone deacetylation. HDAC5 and HDAC9 may also directly interact with MEF2D, SP1, and SP3 proteins and thus inhibit their transactivator activities. At the same time, andrographolide also induces HNC cell death, probably via the necroptosis pathway (Figure 7).

## 3. Discussion

As stated earlier, EBV infection is closely associated with various types of tumors, including B-cell tumors and epithelial tumors. EBV has two infection cycles: lytic and latent. The lytic replication of EBV is associated with viral transmission and plays an important role in the oncogenesis of EBV-associated malignancies [3,11,32]. Therefore, the inhibition of EBV lytic replication could be beneficial for the treatment of EBV-associated diseases.

Our study is the first report pointing to the inhibition of EBV lytic reactivation and the induction of cell death in EBV-positive HNC cell lines via andrographolide treatment. We demonstrated this using proteomic analysis, PPI network prediction, molecular docking, and in vitro study. Andrographolide treatment inhibits EBV lytic replication in P3HR1 cells by inhibiting the expression of lytic proteins, Zta, Rta, and EA-D, and suppressing the promoter activity of Zp and Rp [27]. Consistent with previous reports, we also showed that andrographolide can inhibit EBV lytic reactivation in EBV-positive HNC cell lines via the inhibition of EBV gene expression and EBV particle production. Similarly, other plant-derived compounds, such as glycyrrhizic acid, (-)-epigallocatechin gallate (EGCG), moronic acid, resveratrol, and protoapigenone, can also inhibit EBV lytic replication via the inhibition of the expression of EBV lytic genes through the suppression of Zp and Rp [33,34,35,36,37]. 

As mentioned previously, host factors and TFs, among other factors, play an important role in the regulation of EBV lytic replication. In this study, we also clearly showed that andrographolide treatment inhibited the expression of the transcription factors, MEF2D, SP1, and SP3. Dehydroandrographolide, another major compound extracted from *A. paniculata*, significantly reduces the expression of TFs, c-Fos, c-Jun, and SP1 in SCC9 cells (an oral cancer cell line) [38]. In addition, luteolin, a natural compound extracted from plants, disrupts the binding of SP1 to the Zta and Rta promoters in NA cells during the induction of EBV lytic reactivation via TPA and NaB treatment, as evidenced by chromatin immunoprecipitation (CHIP) [39]. Thus, our findings are consistent with these previous studies in indicating that andrographolide inhibits EBV lytic reactivation by inhibiting the expression of TFs.

Several host factors are involved in the inhibition of EBV lytic replication. The zinc-finger E-box binding factors, ZEB1 and ZEB2, maintain EBV latency by binding to the ZV element on Zp of EBV in EBV-positive B-lymphocyte cell lines [40,41]. In addition, PIAS1 acts synergistically with SAMHD1 to inhibit EBV lytic replication through protein–protein interactions and SUMOylation [42,43]. Our proteome analysis also showed that PIAS1, SAMHD1, ZEB1, and ZEB2, repressors for EBV lytic replication, were significantly increased in the cells treated with andrographolide and were subsequently induced to become lytic with NaB. This result suggests that andrographolide induces the expression of cellular proteins, playing a role in the inhibition of EBV lytic reactivation. 

In addition, the expression of the epigenetic factors DNMT1, DNMT3B, HDAC5, and HDAC9 also increased in the cells treated with the combination of andrographolide and NaB. DNA methyltransferase enzymes, especially DNMT1 and DNMT3B, but not DNMT3A, play a critical role in the maintenance of EBV latency via the restriction of EBNA and LMP expression through DNA methylation in B-lymphocyte cell lines [44,45]. On the other hand, the inhibition of DNMT activities by 5-azacytidine can activate the lytic cycle of EBV by inducing the expression of the lytic gene, BZLF1 [46]. In addition to DNMTs, HDACs can modulate the expression of multiple genes of both the host and EBV by modulating chromosome structure [46,47]. The switching of EBV infection phases from latent to lytic can be induced by stimuli, such as HDAC inhibitors [48,49,50,51,52]. In this study, the expression of HDAC5 and HDAC9 was greater in the cells treated with andrographolide and NaB, suggesting that andrographolide inhibits EBV lytic reactivation via the induction of epigenetic machinery.

Accumulating evidence has demonstrated that MEF2 family members can interact with HDAC family members, especially HDAC4 and HDAC5, to regulate the biological and pathophysiological functions of cells [53,54,55,56,57,58]. Consistent with this finding, by using the STRING database, we also found that HDAC5 and HDAC9 potentially interacted with MEF2D. In addition, HDAC4 and HDAC5 inhibited EBV lytic replication by directly interacting with MEF2 protein that was bound with Zp [59]. 

Previously, we demonstrated that andrographolide suppresses the lytic reactivation of EBV in gastric cancer cell lines by attenuating the expression of host transcription factors, SP1 and MEF2, via epigenetic modifications, especially through the function of HDAC6 and DNMT3A [27,28]. However, in HNC, we demonstrated the inhibitory effect of andrographolide on the EBV lytic replication by modulating the expression of transcription factors SP1, SP3, and MEF2, at least in part, via the epigenetic alterations, especially through the induction of HDAC5 and DNMT3B, suggesting the cell-type-specific mechanism through which andrographolide inhibits the lytic reactivation of EBV. 

Andrographolide treatment can induce cell apoptosis via the activation of various signaling pathways in human cancers, such as skin cancer, colon cancer, cervical cancer, and gastric cancer [60,61,62,63]. In NPC, andrographolide treatment also inhibited cell proliferation and induced cell apoptosis via the activation of various signaling pathways [64,65]. In addition, our previous study also demonstrated a strong cytotoxic effect of andrographolide in gastric cancer cell lines by inducing cell death via apoptosis through the induction of proapoptotic proteins, including BCL2L1, EDOG, HRK, and PUMA [28]. By contrast, in this study, andrographolide exhibited strong cytotoxic effects in EBV-positive HNC cells by inducing cell death. By using bioinformatic tools, we found that the necroptosis-associated proteins were predominant in EBV-positive HNC cells treated with andrographolide and NaB. The key genes of necroptosis, namely RIPK1, RIPK3, and MLKL, were also upregulated. Therefore, this study reveals that a novel mechanism through which andrographolide induces cell death, is, at least in part, necroptosis. 

## 4. Materials and Methods

### 4.1. Cell Lines and Culture Conditions

The NPC cell lines HONE1 and HONE1-EBV (kindly provided by Professor Hironori Yoshiyama, Shimane University, Shimane, Japan) were cultured in an RPMI-1640 medium (Sigma, St. Louis, MO, USA). EBV-negative OSCC cell lines SCC25 and HSC1 (kindly provided by Dr. Tohru Kiyono, National Cancer Center, Tokyo, Japan) and previously established EBV-positive OSCC cell lines SCC25-EBV and HSC1-EBV c [66] were maintained in Dulbecco’s modified Eagle medium/F12 (DMEM/F12; Sigma, St. Louis, MO, USA). All cell lines were supplemented with 10% fetal bovine serum (Gibco, Breda, The Netherlands) and a penicillin–streptomycin solution (Nacalai Tesque Inc., Kyoto, Japan). Cells were cultured at 37 °C in a 5% CO_2_ incubator.

### 4.2. Natural Compounds

The andrographolide compound was prepared as previously described [67] by the Department of Pharmaceutical Sciences, Faculty of Pharmacy, Thammasat University, Thailand. The concentrations of andrographolide used for the treatment of HONE1-EBV, HSC1-EBV, and SCC25-EBV cells were 36.3, 35.0, and 35.0 µM, respectively. 

### 4.3. Proteomic Analysis via Liquid Chromatography with Tandem Mass Spectrometry (LC-MS/MS)

Cells were treated with 0.1% DMSO, or 3 mM NaB, or andrographolide at sub-toxic concentration and incubated for 48 h. In addition, cells were pretreated with andrographolide for 3 h, and the lytic cycle was subsequently induced with NaB and further incubated for 48 h. Protein was extracted from cells using TRIzol™ reagent (Invitrogen, Carlsbad, CA, USA) according to the manufacturer’s instructions. The concentration of total proteins was determined using a Bio-Rad protein-assay dye reagent concentration kit. To prepare protein samples for mass spectrometry, tryptic in-gel digestion was performed as described [68]. 

The trypsin-digested peptides were introduced into LC-MS/MS using an Ultimate3000 Nano/Capillary LC System (Thermo Scientific, Waltham, MA, USA) coupled with a hybrid quadrupole Q-Tof Impact II™ (Bruker, Billerica, MA, USA) equipped with a nano-captive spray ion source. The proteomic analysis via LC-MS/MS was performed as previously described [68]. 

### 4.4. Data Processing and Analysis

To identify and quantify peptides and proteins, MaxQuant version 1.6.5.0 was used. Our data were run against the UniProtKB Human reference database (accessed in October 2020) using the Andromeda search engine. Fragment ion tolerance was set to 20 ppm, and the option of matching between runs (0.4 min match time window) was enabled. Trypsin was specified as the proteolytic enzyme with up to two missed cleavage sites authorized. The N-terminal protein acetylation and oxidation of methionine were set as variable modifications, while carbamidomethylated cysteine was set as a fixed modification. Exploration outcomes were determined for a minimum length of seven amino acids (1% peptide and protein FDR). If a minimum of two peptides was compared between sample groups, the label-free protein quantification (LFQ) was calculated. The match-between-runs feature of MaxQuant was only used in experimental replicates.

The functional annotation (GO) of differentially expressed proteins was performed using the DAVID v6.8 web tool (https://david.ncifcrf.gov/, accessed on 3 March 2021). Differentially expressed proteins were analyzed in terms of two factors: biological process and molecular function. Categories with a *p* value > 0.05 were considered significant, and the top ten categories are presented in this paper. The KEGG pathway analysis was conducted to identify the biological function of differentially expressed proteins. Differentially expressed proteins that were found among the different treatments were identified using the jvenn web tool (http://jvenn.toulouse.inra.fr, accessed on 21 March 2021).

To construct a PPI network, STRING version 11.5 (https://string-db.org/, accessed on 9 September 2021) was used to explore the possible interactions of differentially expressed proteins at the protein level [69]. A PPI score of >0.4 was considered significant. The PPI networks were visualized using Cytoscape software (version 3.8.2) [70], and *p* < 0.05 was considered a statistically significant difference. The interactions of HDAC5 and HDAC9 with TFs were predicted by using the PSOPIA web tool [71]. 

To further confirm protein–protein interactions, molecular modeling and molecular docking simulation were performed. The sequences of candidate proteins, including HDAC5 (Uniprot ID: Q9UQL6), HDAC9 (Uniprot ID: Q9UKV0), MEF2D (Uniprot ID: Q14814), SP1 (Uniprot ID: P08047), and SP3 (Uniprot ID: Q02447), were submitted to SWISS-MODEL, https://swissmodel.expasy.org/ (accessed on 24 November 2021) [72], a protein prediction software, to build the putative 3D structures. The stereochemistry of proteins was evaluated using Ramachandran plot analysis (https://zlab.umassmed.edu/bu/rama/, accessed on 28 November 2021). All ionizable amino acid residues in the predicted models were protonated at pH 7.4 using H++ 1.0 (http://biophysics.cs.vt.edu/, accessed on 28 November 2021). Molecular docking simulation was performed using the web-based service ClusPro [73] to analyze the interactions among the candidate proteins. The PyMOL and Discovery Studio 2021 software version 21.1 were used for the visualization of 3D models and molecular docking. The coefficient weights of protein binding were calculated as follows: E = 0.40E_rep_ + (−0.40)E_att_ + 600E_elec_ + 1.00E_DARS_ [74].

### 4.5. Quantification of Gene Expression via qRT-PCR

The total RNA was extracted from cells using TRIzol™ reagent (Invitrogen, Carlsbad, CA, USA), according to the manufacturer’s instructions, and 1 µg of RNA was used to synthesize cDNA using a RevertAid First Strand cDNA Synthesis Kit (Thermo Scientific, Waltham, MA, USA) according to the manufacturer’s instructions. Gene expression was quantified with a qRT-PCR assay using SsoAdvanced^TM^ SYBR^®^ Green SuperMix (Bio-Rad, Hercules, CA, USA) in the QuantStudio 6 Flex Real-Time PCR System (Applied Biosystems, Foster City, CA, USA). Glyceraldehyde 3-phosphate dehydrogenase (GAPDH) was used as an internal control. The relative mRNA expression level was quantified using the 2^−ΔΔCT^ method. The primers used in the present study are listed in Table 2.

### 4.6. Quantification of EBV Genome’s Copy Number via qPCR

The EBV genome’s copy number was estimated with qPCR targeting the EBNA1 gene. Briefly, genomic DNA was extracted from the cell-culture supernatant using the lysis buffer–proteinase K method [75]. Calibration curves were generated in parallel with each analysis using sequential dilutions of DNA from the pGEM-T-EBNA1 plasmid. The copy number of the EBV genome in cells was analyzed with a calibration curve using CT values that were obtained from the samples. The CT values were used to calculate the EBV genome’s copy number from a linear regression equation. The results are expressed as the mean (based on three independent experiments) of EBV copies/50 ng of host genomic DNA.

### 4.7. Determination of Cell Cytotoxicity Using CCK-8

Cell cytotoxicity was determined using CCK-8 (DOJINDO, Kumamoto, Japan). Cells were seeded into a 96-well plate at 5000 cells/well and preincubated at 37 °C under 5% CO_2_ for 24 h. The cells were treated with DMSO, NaB, or andrographolide. In addition, for the treatment using the combination of NaB and andrographolide, the cells were pretreated with andrographolide for 3h, followed by treatment with NaB, to induce the lytic cycle. Subsequently, the cells were further incubated for 24 and 48 h. Then, the cells were incubated with 10 µL/well of CCK-8 solution for 1–4 h, and absorbance was measured at 450 nm using a Varioskan LUX Multimode Microplate Reader (Thermo Scientific, Waltham, MA, USA).

### 4.8. Quantification of Cell Death via Flow Cytometry

The dead cells were quantified using the Dead Cell Apoptosis Kit with Annexin V Alexa Fluor™ 488 and Propidium Iodide (PI) Kit (Life Technologies, Carlsbad, CA, USA). Cells were plated in 6-well plates at a seeding density of 2.5 × 10^5^ cells/well and preincubated at 37 °C under 5% CO_2_ until 90% confluent. The cells were then treated with DMSO, NaB, or andrographolide, or they were pretreated with andrographolide followed by NaB to induce the lytic cycle and further incubated for 18 h. The cells were stained with Annexin V and PI at room temperature for 15 min and further analyzed via flow cytometry using a BD FACSCanto™ II Cell Analyzer (BD bioscience, Franklin Lakes, NJ, USA). Data were analyzed with FlowJo software version 9 (Flowjo, Treestar Inc., Ashland, OR, USA). Staurosporine treatment was used as a positive control for cell apoptosis. The cells stained by both Annexin V and PI were considered late apoptotic cells, those only positive for Annexin V staining were considered early apoptotic cells, and those only positive for PI staining were considered necrotic cells.

### 4.9. Statistical Analysis

GraphPad Prism (https://www.graphpad.com/, accessed on 28 November 2021) (GraphPad Software Inc., San Diego, CA, USA) was used for all data analysis. Mann–Whitney tests were used to test whether there was a difference between two independent groups, expressed as mean ± standard deviation (SD). All experiments were repeated three times. A probability (*p*) value of <0.05 was considered statistically significant.

## 5. Conclusions

In the present study, we have demonstrated for the first time that andrographolide inhibits EBV lytic reactivation in HNC cell lines via the inhibition of TF expression. The expression of TFs is probably regulated through epigenetic mechanisms, such as DNA methylation and histone modifications. Concurrently, andrographolide also induces cell death via necroptosis through the upregulation of key mediators, including genes encoding RIPK1, RIPK3, and MLKL.

## Figures and Tables

**Table 1:** Prediction of protein–protein interactions (PPIs).

Protein	Protein	AODE Scores			
		S_seq_ *	S_dom_ *	S_net_ *	All *
HDAC5	MEF2D	0.9962	0.9965	0.8351	0.9991
	SP1	0.813	0.5146	0.8351	0.9011
	SP3	0.813	0.5146	0.8351	0.9011
	SAMHD1	0.3537	0.5146	0.0000	0.3197
HDAC9	MEF2D	0.9962	0.9965	0.8351	0.9991
	SP1	0.813	0.5146	0.8351	0.9011
	SP3	0.813	0.5146	0.8351	0.9011
	SAMHD1	0.3537	0.5146	0.0000	0.3197

* S_seq_ is a score assigned using sequence similarities to a known interacting protein pair. S_dom_ is a score assigned using the statistical propensities of domain–domain interactions. S_net_ is a score assigned using a sum of edge weights along the shortest path between homologous proteins in a PPI network. ALL is a score assigned using all three features: S_seq_, S_dom_, and S_net_.

**Table 2:** Primer sequences.

Gene	Forward (5′-3′)	Reverse (5′-3′)
*BMRF1*	ACCTGCCGTTGGATCTTAGTG	GGCGTTGTTGGAGTCCTGTG
*BRLF1*	TGTTTCAACCGCTCCGACTG	GGGTTATGTCGGAGACTGGG
*BZLF1*	TGTTTCAACCGCTCCGACTG	GGGTTATGTCGGAGACTGGG
*EBNA1*	CCACAATGTCGTCTTACACC	ATAACAGACAATGGACTCCCT
*HDAC5*	CCTCAACCATTCCCTCCCAC	GTTCAGAGGCTGTTTTGCGG
*HDAC9*	CCCCTGCTGCCTCTGTTTTA	GGAATTGCCACAAACGCACT
*GAPDH*	TCATCAGCAATGCCTCCTGCA	TGGGTGGCAGTGATGGCA
*MEF2D*	CATGCCCACTGCCTACAACA	TGACATTGCCTAGCGACAGC
*MLKL*	CGGCCCTCTGTGGATGAAAT	GCCTCTCCCAGCTTCTTGTC
*RIPK1*	CGACCTTCTGAGCAGCTTGA	TCTGAATGCTCTGAGGCAGC
*RIPK3*	CATGGAGAACGGCTCCTTGT	GGTTCTGGTCGTGCAGGTAA
*SP1*	CTGTGATACGGATCAGAAACCG	TCCACCAAACAATAAAGAGTGCT
*SP3*	CAGAAAGGGTGGGCCTTGAA	GCCATCTGTTAAGAGGGCGT

## Data Availability

The published article includes all the datasets generated or analyzed during this study.

## Supplementary Materials

The supporting information can be downloaded at: https://www.mdpi.com/article/10.3390/ijms242115867/s1.
