# Supplementary material for: The Dual Functions of Andrographolide in the Epstein–Barr Virus-Positive Head-and-Neck Cancer Cells: The Inhibition of Lytic Reactivation of the Epstein–Barr Virus and the Induction of Cell Death
Source: Int J Mol Sci. 2023 Nov 1;24(21):15867. doi: 10.3390/ijms242115867 (PMC10648111; doi:10.3390/ijms242115867)
Supplement: Supplementary file 1 [file ijms-24-15867-s001.zip › Table S1.pdf]

Table S1. The interaction of HDAC5 either with MEF2D, SP1 or SP3 via hydrogen bond formations.

No.	HDAC5-MEF2D	HDAC5:SP1	HDAC5:SP3
1	ARG24:HH11-GLU34:OE1	ARG654:HH22-ASP789:OD2	ASP879:OD2-LYS5:HZ2
2	ARG24:HH21-GLU34:OE1	LYS665:HZ1-ASP787:OD1	ASP914:OD1-ARG3:HH11
3	LYS25:HZ1-GLU77:OE1	LYS665:HZ3-ASP787:OD2	ASP914:OD2-ARG3:HH12
4	LYS25:HZ3-GLU77:OE2	HIS702:HD1-HIS646:O	ASP739:OD2-LYS4:HZ3
5	LYS53:HZ1-GLU74:OE1	HIS704:HN-ARG648:O	GLU847:OE1-LYS30:HZ3
6	LYS53:HZ3-GLU74:OE2	SER788:HG-HIS676:NE2	LYS999:HZ1-ARG89:O
7	ASP789:OD1-ARG10:HH12	LEU1008:HN-HIS650:O	LYS999:HZ2-LYS91:O
8	ASP789:OD2-ARG17:HH21	GLY699:O-LYS635:HZ1	LYS999:HZ3-LYS90:O
9	GLU34:OE1-ARG24:HH11	HIS702:NE2-LYS635:HZ3	LEU878:O-GLY2:HT1
10	GLU34:OE1-ARG24:HH21	ASN700:OD1-TYR637:HH	ASN992:OD1-LYS5:HZ1
11	GLU77:OE1-LYS25:HZ1	THR701:O-TRP649:HE1	GLY876:O-LYS5:HZ2
12	GLU77:OE2-LYS25:HZ3	HIS707:NE2-HIS650:HD1	GLU847:O-LYS91:HZ3
13	GLU74:OE1-LYS53:HZ1	ASN899:OD1-SER661:HG	PRO922:O-GLY2:HN1
14	GLU74:OE2-LYS53:HZ3	SER788:OG-LYS665:HZ2	HIS782:NE2-ARG3:HH21
15	SER788:HG-GLU14:OE2	PRO972:O-ARG666:HH12	HIS782:NE2-ARG3:HH22
16	ILE11:HN-LEU38:O	PRO972:O-ARG666:HH21	HIS783:NE2-ARG3:HH22
17	GLU42:HN-PHE48:O	HIS872:NE2-ARG666:HH22	ASP739:O-LYS4:HZ3
18	ALA44:HN-ILE46:O	VAL703:O-THR668:HG1	SER728:O-THR12:HG1
19	ILE46:HN-ALA44:O	PRO780:O-ARG669:HH22	VAL736:O-ARG15:HH12
20	PHE48:HN-GLU42:O	PRO780:O-ARG675:HH11	VAL736:O-ARG15:HH22
21	HIS50:HN-ASP40:O	VAL778:O-ARG697:HH12	ASP737:OD1-ASN16:HD22
22	HIS50:HD1-CYS39:O	VAL778:O-ARG697:HH22	ASP846:O-LYS30:HZ2
23	HIS50:HD1-ASP40:O	LEU779:O-ARG697:HH22	ASN849:OD1-LYS30:HZ2
24	LYS53:HZ1-PRO75:O	HIS702:HE1-HIS650:NE2	ASP846:O-LYS30:HZ3
25	LYS53:HZ2-TYR72:OH	PRO972:HD1-ARG654:O	PRO655:HD1-ARG3:O
26	GLN56:HN-GLU77:O	ASP1007:HA-THR651:O	PRO880:HD2-ARG3:O
27	GLN56:HE21-SER78:OG	HIS704:O-TRP649:HA	LEU923:HA-GLY2:O
28	GLN56:HE22-TYR69:OH	HIS704:O-TRP649:HD1	ASP879:OD1-LYS5:HA
29	ALA58:HN-ARG79:O	ASN899:OD1-SER661:HB2	ASN992:OD1-LYS5:HE1
30	LYS68:HZ3-ASN52:OD1		GLU847:O-LYS91:HE2
31	TYR69:HH-ASP63:OD1		HIS822:NE2-GLY2:HA1
32	GLU77:HN-LEU54:O		HIS956:NE2-ARG3:HD1
33	ARG79:HN-GLN56:O		ASP739:O-LYS4:HE1
34	THR80:HG1-ALA58:O		GLU847:OE2-LYS30:HE2
35	THR80:HG1-SER59:O		
36	ASN81:HN-ALA58:O		
37	ASN81:HN-SER59:O		
38	ASN81:HD22-SER36:OG		
39	VAL928:O-ARG3:HH11		
40	VAL928:O-ARG3:HH12		
41	SER977:OG-LYS4:HZ1		
42	TYR976:O-LYS4:HZ2		
43	GLY968:O-GLN7:HN		
44	ASP1007:OD1-GLN7:HE21		
45	HIS1006:O-GLN9:HE22		
46	VAL37:O-ARG10:HN		
47	VAL703:O-ARG10:HE		
48	ASP40:OD1-ARG10:HE		
49	SER788:O-ARG10:HH11		
50	SER788:O-ARG10:HH21		
51	LEU38:O-ILE11:HN		
52	GLY898:O-ARG15:HH11		
53	ASN899:OD1-ARG15:HH12		

54	GLY898:O-ARG15:HH21		
55	PHE900:O-ARG15:HH21		
56	GLU34:O-ARG24:HH12		
57	ASN81:OD1-SER36:HG		
58	PHE48:O-GLU42:HN		
59	ILE46:O-ALA44:HN		
60	ALA44:O-ILE46:HN		
61	GLU42:O-PHE48:HN		
62	CYS39:O- HIS50:HN		
63	CYS39:O-HIS50:HD1		
64	PRO75:O-LYS53:HZ1		
65	TYR72:OH-LYS53:HZ2		
66	GLU77:O-GLN56:HN		
67	SER78:OG-GLN56:HE21		
68	TYR69:OH-GLN56:HE22		
69	ARG79:O-ALA58:HN		
70	ASN52:OD1-LYS68:HZ3		
71	ASP63:OD1-TYR69:HH		
72	LEU54:O-GLU77:HN		
73	GLN56:O-ARG79:HN		
74	THR60:O-THR80:HG1		
75	SER59:O-ASN81:HN		
76	SER59:O-ASN81:HD21		
77	SER36:OG-ASN81:HD22		
78	PRO705:HA-GLN9:OE1		
79	SER788:HB1-HIS50:NE2		
80	PHE901:HA-ASP13:OD1		
81	GLY968:HA1-LYS5:O		
82	ARG10:HA-VAL37:O		
83	ARG10:HA-LEU38:O		
84	CYS41:HA-PHE48:O		
85	ILE43:HA-ILE46:O		
86	LEU45:HA-ALA44:O		
87	HIS50:HE1-CYS39:O		
88	LYS53:HE2-PRO75:O		
89	PHE55:HA-GLU77:OE2		
90	PHE55:HA-GLU77:O		
91	SER78:HA-GLN56:O		
92	THR80:HA-ALA58:O		
93	SER977:OG-LYS4:HE1		
94	GLY968:O-ILE6:HA		
95	VAL37:O-ARG10:HA		
96	LEU38:O-ARG10:HA		
97	VAL703:O-ARG10:HD1		
98	ASP789:OD1-ARG10:HD2		
99	ASP789:OD2-THR12:HA		
100	ASN899:OD1-ARG15:HD1		
101	GLU34:O-ARG24:HD2		
102	ASN81:OD1-TYR33:HA		
103	GLN9:O-LEU38:HA		
104	PHE48:O-CYS41:HA		
105	ILE46:O-ILE43:HA		
106	ALA44:O-LEU45:HA		
107	ASP40:O-HIS50:HA		
108	PRO75:O-LYS53:HE2		
109	GLU77:O-PHE55:HA		
110	ARG79:O-TYR57:HA		

111	ASN81:OD1-SER59:HA		
112	ASN52:OD1-LYS68:HE1		
113	GLN56:O-SER78:HA		
114	ALA58:O-THR80:HA		
