# Supplementary material for: The Dual Functions of Andrographolide in the Epstein–Barr Virus-Positive Head-and-Neck Cancer Cells: The Inhibition of Lytic Reactivation of the Epstein–Barr Virus and the Induction of Cell Death
Source: Int J Mol Sci. 2023 Nov 1;24(21):15867. doi: 10.3390/ijms242115867 (PMC10648111; doi:10.3390/ijms242115867)
Supplement: Supplementary file 1 [file ijms-24-15867-s001.zip › Table S2.pdf]

Table S2. The interaction of HDAC9 either with MEF2D, SP1 or SP3 via hydrogen bond formations.

No.	HDAC9:MEF2D	HDAC9:SP1	HDAC9:SP3
1	ASP879:OD2-LYS5:HZ2	GLU785:OE2-ARG666:HH12	ASP737:OD1-LYS660:HZ1
2	ASP914:OD1-ARG3:HH11	ASP721:OD2-LYS703:HZ3	ASP737:OD2-LYS660HZ3
3	ASP914:OD2-ARG3:HH12	SER738:HN-THR668:OG1	LYS724:HZ2-THR674:O
4	ASP739:OD2-LYS4:HZ3	THR653:O-HIS650:HD1	SER728:HG-ASP694:OD2
5	GLU847:OE1-LYS30:HZ3	ALA789:O-ARG654:HH11	PHE851:HN-SER646:O
6	LYS999:HZ1-ARG89:O	ALA789:O-ARG654:HH22	GLU786:OE2-TYR657:HH
7	LYS999:HZ2-LYS91:O	PHE725:O-LYS665:HZ1	ALA789:O-LYS660:HZ1
8	LYS999:HZ3-LYS90:O	VAL736:O-LYS665:HZ3	VAL736:O-LYS660:HZ2
9	LEU878:O-GLY2:HT1	MET790:SD-ARG666:HH22	PRO852:O-ARG661:HH21
10	ASN992:OD1-LYS5:HZ1	SER738:OG-ARG669:HH21	SER738:OG-THR663:HG1
11	GLY876:O-LYS5:HZ2	GLN723:O-ARG697:HH12	LYS724:O-THR674:HG1
12	GLU847:O-LYS91:HZ3	GLN723:OE1-LYS703:HZ1	ASP720:O-LYS688:HZ1
13	PRO922:O-GLY2:HN1	THR652:HB-GLY632:O	GLN723:O-ARG692:HH11
14	HIS782:NE2-ARG3:HH21	SER738:HB2-THR668:OG1	GLN723:O-ARG692:HH22
15	HIS782:NE2-ARG3:HH22	THR652:O-GLY632:HA2	ASP721:OD2-HIS695:HD1
16	HIS783:NE2-ARG3:HH22	ASP739:OD1-TRP649:HA	SER728:OG-LYS698:HZ1
17	ASP739:O-LYS4:HZ3	SER738:O-TRP649:HD1	GLN723:OE1-LYS698:HZ3
18	SER728:O-THR12:HG1	THR653:O-HIS650:HE1	LYS724:HE2-THR674:O
19	VAL736:O-ARG15:HH12	ASP739:OD2-GLY652:HA1	SER738:HB2-GLU667:OE1
20	VAL736:O-ARG15:HH22	ASP737:OD1-ARG654:HD1	PRO922:HA-HIS645:O
21	ASP737:OD1-ASN16:HD22	THR788:OG1-GLY664:HA2	ALA789:O-LYS660:HE1
22	ASP846:O-LYS30:HZ2	VAL736:O-LYS665:HE1	PHE725:O-HIS671:HE1
23	ASN849:OD1-LYS30:HZ2	SER738:OG-ARG669:HD2	VAL736:O-HIS671:HE1
24	ASP846:O-LYS30:HZ3		ASP720:OD2-LYS679:HE1
25	PRO655:HD1-ARG3:O		GLN723:O-HIS695:HE1
26	PRO880:HD2-ARG3:O		
27	LEU923:HA-GLY2:O		
28	ASP879:OD1-LYS5:HA		
29	ASN992:OD1-LYS5:HE1		
30	GLU847:O-LYS91:HE2		
31	HIS822:NE2-GLY2:HA1		
32	HIS956:NE2-ARG3:HD1		
33	ASP739:O-LYS4:HE1		
34	GLU847:OE2-LYS30:HE2		
